# Clinical trials of artesunate plus sulfadoxine-pyrimethamine for *Plasmodium falciparum* malaria in Afghanistan: maintained efficacy a decade after introduction

**DOI:** 10.1186/s12936-016-1167-z

**Published:** 2016-02-25

**Authors:** Ghulam Rahim Awab, Mallika Imwong, Sasithon Pukrittayakamee, Fazel Alim, Warunee Hanpithakpong, Joel Tarning, Arjen M. Dondorp, Nicholas P. J. Day, Nicholas J. White, Charles J. Woodrow

**Affiliations:** Department of Clinical Tropical Medicine, Faculty of Tropical Medicine, Mahidol University, Bangkok, Thailand; Ministry of Public Health, Kabul, Afghanistan; Department of Molecular Tropical Medicine and Genetics, Faculty of Tropical Medicine, Mahidol University, Bangkok, Thailand; Mahidol-Oxford Tropical Medicine Research Unit (MORU), Faculty of Tropical Medicine, Mahidol University, Bangkok, Thailand; Centre for Tropical Medicine and Global Health, Nuffield Department of Medicine, University of Oxford, Oxford, UK

**Keywords:** Malaria, Falciparum, Afghanistan, Artesunate, Sulphadoxine, Pyrimethamine, Dihydrofolate reductase, Dihydropteroate synthase, Piperaquine, Kelch

## Abstract

**Background:**

Combination therapy with artesunate plus sulfadoxine-pyrimethamine (SP) was adopted as recommended treatment for *Plasmodium falciparum* infection in Afghanistan in 2003.

**Methods:**

A series of prospective clinical studies examining the efficacy of artesunate plus sulfadoxine-pyrimethamine (AS + SP) against *P. falciparum* were undertaken in sentinel sites in Afghanistan from 2007 to 2014, accompanied by relevant molecular studies. The first study was a randomized trial of AS + SP versus dihydroartemisinin-piperaquine, while two subsequent studies were standard therapeutic efficacy studies of AS + SP.

**Results:**

Three hundred and three patients were enrolled across four provinces in the north and east of the country. Curative efficacy was high in all the trials, with an adequate clinical and parasitological response (ACPR) of more than 95 % in all groups and trial stages. Genotyping for drug-resistance alleles at *dhfr* indicated fixation of the S108 N mutation and a prevalence of the C59R mutation of approximately 95 % across all sites. Other mutations in *dhfr* and *dhps* remained rare or absent entirely, although five isolates from the first trial carried the *dhps* triple mutant SGEGA haplotype. In the last study undertaken in 2012–2014 the K13 artemisinin resistance marker was examined; only two of 60 successfully sequenced samples carried a K13-propeller mutation.

**Conclusions:**

These data confirm maintained efficacy 10 years after introduction of artesunate plus SP as combination treatment of *P. falciparum* in Afghanistan. The molecular data indicate that despite a substantial fall in incidence, resistance has not developed to artemisinins, or intensified to the ACT partner drug components.

*Trial Registration*http://www.clinicaltrials.gov/ct NCT00682578, NCT01115439 and NCT01707199

## Background

*Plasmodium falciparum* transmission occurs at its most northerly latitudes in Afghanistan, where the distribution of cases is determined by the physical geography of the country, malaria being confined to lower lying areas with sufficient rainfall for mosquito survival. These consist of the northern plains [1], the Jalalabad basin to the east (bordering Pakistan), and river valleys that fringe the central mountains to the west and south [2]. The central area of Afghanistan, occupied by the western end of the Hindu Kush mountain range, is relatively free of malaria [3, 4]. The incidence of clinical cases peaks in November [5]. Following falls in incidence, northern areas are now preparing for malaria elimination in line with neighbouring countries (Turkmenistan, Uzbekistan and Tajikistan).

Chloroquine-resistant *P. falciparum* was evident in Afghanistan and Pakistan by the 1990s [6–9] and the combination of artesunate with amodiaquine also proved to have low efficacy [7] consistent with spread of the SVMNT *pfcrt* haplotype [10]. However, sulfadoxine-pyrimethamine (SP) retained reasonable efficacy in eastern [7] and northern Afghanistan (Kunduz province, 2002–2003) [11], findings which suggested that AS + SP might be an efficacious therapy for falciparum malaria in the region. Combination therapy with AS + SP was adopted as recommended treatment for *P. falciparum* infection in a number of south Asian countries, becoming first-line treatment in Afghanistan (2003), Iran (2006), Pakistan (2007) and India (2007).

This paper describes a series of therapeutic efficacy studies undertaken to examine the efficacy of AS + SP against *P. falciparum* in sentinel sites in Afghanistan from 2007 to 2014, accompanied by relevant molecular studies. These confirm maintained efficacy of the combination against *P. falciparum* with no evidence for increase in prevalence of molecular markers of artemisinin and SP resistance.

## Methods

### Sites

Studies were undertaken at, or within 20 km of, four provincial control centres, two located to the north and two to the south and east of the Hindu Kush mountains. Jalalabad, capital of Nangarhar province, is a referral centre for the whole eastern region and lies adjacent to the Pakistani border where population movement in both directions takes place. The population is multi-ethnic with Pashtoon being the dominant group. The province as a whole is generally semiarid but where possible rice is cultivated. Asadabad is the capital city of Kunar province, a largely mountainous area bordering Pakistan, with which there is uncontrolled movement via unofficial borders. Taloqan is the capital of the north-east province of Takhar with a population that is mainly Tajik with significant proportions of Uzbek and Pashtoon. Rice is cultivated on the plain and malaria is a well-known health problem [12]. Maimana is the capital city of the north-west province of Faryab bordering Turkmenistan, with which there is no open border. The population is mainly Uzbek and Turkmen and the region is semi-arid.

### Participants

Trial 1 was a prospective, open label, parallel group randomized trial with a 1:1 allocation ratio of AS + SP to dihydroartemisinin-piperaquine (DP) undertaken in all four centres. Trials 2 and 3 were therapeutic single-arm efficacy studies of AS + SP undertaken in Jalalabad (Nangarhar province), and Asadabad (Kunar province) respectively. In all trials eligible participants were adults and children over 6 months old presenting with symptomatic, uncomplicated, microscopically confirmed monoinfection with asexual stages of *P. falciparum*. Additional study inclusion criteria were the ability to swallow oral medication, ability and willingness to comply with the study protocol for the duration of the study and written informed consent (from a parent or guardian in the case of children under 18 years of age). In trial 1 there was no lower limit of asexual parasitaemia and patients with parasitemia of more than 100,000/µL were excluded. In trials 2 and 3 only patients with 500–150,000/µL asexual forms were included.

In all trials exclusion criteria included the presence of general danger signs in children aged under 5 years or signs of severe falciparum malaria according to the definitions of WHO [13], mixed malaria infection, enrollment in any other investigational study in the previous month, known underlying chronic disease requiring treatment, a history of hypersensitivity reactions or contraindications to any of the study medications and pregnancy. Pregnancy was excluded via urine dipstick testing, although unmarried females over 12 years of age were also included without a urine test given the cultural sensitivity of pregnancy testing in this group. In trial 1, treatment with antimalarials in the past month was an additional exclusion criterion. In trials 2 and 3, additional exclusion criteria were severe malnutrition (defined as a child whose growth standard is below −3 z-score, has symmetrical oedema involving at least the feet or a mid-upper arm circumference < 110 mm), presence of febrile conditions due to diseases other than malaria and regular medication potentially interfering with antimalarial pharmacokinetics.

### Trial methodology

Standard case record forms were used to record demographic information, details of symptoms and their duration, and previous antimalarial medications. Clinical examination findings and vital signs were documented, including axillary temperature measured with a digital thermometer. Blood samples were obtained for haemoglobin measurement and Giemsa-staining of thick and thin blood films to confirm parasite species and parasitaemia by a standard approximation method (40 × number of parasites per 200 white blood cells on the thick film). Slide examination results were considered to be negative after examination of 200 oil fields of the thick film. 20–100 µl baseline blood was collected on filter papers for subsequent confirmation of species by PCR and testing of drug resistance markers.

### Interventions

For patients receiving AS + SP, each drug was dosed to the nearest half tablet, determined from a dosing chart [14] and broadly equivalent to a daily dose of 4 mg artesunate/kg (once daily for 3 days) and a single dose of 25 mg sulphadoxine/kg on day 0. Trial 1 used 50 mg artesunate tablets from Guilin Pharmaceutical, China and SP (500/25 mg tablets) from Roche, Switzerland. Trial 2 used Guilin artesunate and SP (500/25 mg tablets) from Durbin Pharmaceuticals. Trial 3 used blister packs (Artecospe® from Guilin Pharmaceutical Co. Ltd., China or Artesul from Zafa) containing SP tablets (500/25 mg) and 50 or 100 mg artesunate tablets. In Trials 2 and 3, drugs were provided by WHO via the Afghanistan National Malaria Control Program after quality control in a pre-qualified laboratory. Primaquine was not administered in any of the studies.

Patients receiving DP (trial 1 only) received Duo-Cotecxin® (Holleypharm, China) in which each co-formulated tablet contained 40 mg dihydroartemisinin and 320 mg piperaquine phosphate. The target total dose of 6/48 mg/kg was made up of three divided doses given on a daily basis. According to the body weight, individual doses were rounded to the nearest quarter tablet.

All doses of medicine were administered under the supervision of a qualified member of the staff designated by the principal investigator. The study patients were observed for 30 min after medicine administration for adverse reactions and/or vomiting. Patients who vomited during this observation period were re-treated with the same dose of medicine and observed for an additional 30 min. Patients vomiting again were to be given parenteral (and subsequently oral) therapy with quinine sulphate (10 mg base per kg body weight three times per day), along with once daily tetracycline (clindamycin in children) according to national malaria treatment guidelines, and withdrawn from further study.

### Follow-up

Subjects were seen on days 0–3 inclusive and weekly to 56 days (trial 1) or 42 days (trials 2 and 3). Patients were also requested to attend if they felt unwell on any other day during follow-up. At each follow up visit a clinical assessment was carried out and the patient was asked about adverse events by the use of a standard symptom questionnaire. Thick and thin blood slides were examined and haemoglobin was measured. Other tests such as peripheral white blood cell count and urine examination were undertaken as indicated to rule out other conditions and investigate possible adverse effects of drugs. Patients were censored from the analysis if there was stated withdrawal of consent at any stage, persistent vomiting during the acute phase (necessitating parenteral treatment) or occurrence during the follow-up of concomitant disease that would interfere with a clear classification of the treatment outcome (including infection with *P. vivax*). Patients failing to attend follow-up visits received a visit at home and were asked to re-attend the centre for scheduled assessments. Transportation was provided for all follow-up visits for all subjects. All enrolled patients were given one long-lasting insecticide-treated bed net.

Patients with recurrence of *P. falciparum* had capillary blood collected on filter paper for PCR analysis and were treated with oral quinine and tetracycline (clindamycin in children) for 7 days with standard follow up.

### Outcomes

The primary outcome was the proportion of patients with PCR-corrected adequate clinical and parasitological response (ACPR) at day 42 [15]. Secondary outcomes were the proportion of patients with PCR-corrected ACPR at day 28, crude or PCR-uncorrected ACPR, early treatment failure, parasite, fever and gametocyte clearance times, haemoglobin recovery and the rate and severity of adverse events.

### Sample size

In trial 1 the aim was to examine whether DP was as effective as AS + SP via a non-inferiority design, given the relatively high predicted efficacy of AS + SP [16]; the non-inferiority margin (Δ) was set at 5 %. Sample size was 550 patients (275 per arm), calculated assuming a 95 % cure rate with AS + SP, a one-sided alpha of 0.05 and 80 % power.

Trials 2 and 3 were single arm monitoring studies and a sample size of 100 was chosen in each case consistent with WHO guidelines for therapeutic efficacy studies [15].

### Randomisation and blinding (trial 1)

Patients were allocated to the two treatment arms based on a randomization list created using Stata 9.0 statistical software (StataCorp, College Station, TX) with a 1:1 allocation using a block size of 20 that was produced and held independently of the field teams by a statistician. The individual treatment allocations were kept in sequentially numbered, opaque, sealed envelopes and opened by the relevant field worker only after participant enrolment by the field team. Patients and clinical field workers were not blinded to the treatment arm after allocation. Laboratory workers assessing blood films and genotyping were not informed of the treatment arm.

### Statistical methods

Data were entered into Microsoft Access or the WHO Study Information Sheet for calculation of baseline characteristics. Subsequent analyses were conducted using STATA (versions 10–12, Statcorp, USA) and GraphPad Prism (GraphPad, USA). The Student’s t test, Mann–Whitney U and Chi squared (or Fisher’s exact) tests were used for comparison of baseline variables between the two treatment arms of trial 1 and Wilson’s test was used for calculation of confidence intervals for ACPR.

### Pharmacological studies

Day 7 blood samples were collected for drug measurements in Nangarhar and Kunar provinces in year 3 of trial 1, during the period September to December 2009. Approximately 100 µl capillary blood was collected onto Whatman 3MM filter papers and stored individually with silica gel for transportation to the Department of Clinical Pharmacology at MORU. Piperaquine concentrations (DP arm), adjusted for estimated sample volume, were determined by liquid chromatography and tandem mass spectrometry as described previously [17, 18]. Sulphadoxine concentrations (AS + SP studies) were determined by high performance liquid chromatography coupling with ultraviolet detector. This method is based on the method developed by Green et al. [19] with a slightly extended calibration range at both ends. Quality control samples (low, medium and high concentration) were analysed in triplicates within each batch to ensure accuracy and precision during routine drug measurements of clinical samples. Total accuracy and precision were below 7 % CV for all quality control samples. The lower limit of quantification was set to 3 ng/mL for piperaquine and 2 µg/mL for sulphadoxine.

### Genotyping of baseline and recurrent infections

Blood samples were collected on filter paper (Whatman 3MM) at enrollment and at any visit where parasites were observed after day 7. Each filter paper was dried and stored individually in a plastic bag containing silica gel. All filter papers were subsequently transported to the Faculty of Tropical Medicine, Mahidol University. Parasite DNA from dried blood spots was extracted via the QIAmp DNA Mini kit using the standard protocol and stored at −20 °C until use. Nested PCR was performed to detect *P. falciparum* according to established methods. In order to differentiate a recrudescence from a reinfection, a genotype analysis was conducted based on the extensive genetic diversity in the polymorphic genes *msp1*, *msp2* and *glurp*. The genotypic profiles of pre- and post-treatment strains were compared by standard methods [20].

PCR–RFLP and sequencing was undertaken for baseline samples in trial 1 examining *pfdhr*, *pfdhps*, *pfcrt* and *pfmdr1* single nucleotide polymorphisms (SNPs) and *pfmdr1* copy number assessment using previously described techniques [21, 22]; these data have already been published [23]. In trials 2 and 3 SNPs in *pfdhr* and *pfdhps* alone were examined and in trial 3 the artemisinin resistance marker K13 propeller [24] was sequenced using established techniques [25]. Heterozygous samples with evidence of both wild-type and mutant sequence were counted as mutants in overall calculations.

### Ethical approval

All trials were approved by the Oxford Tropical Research Ethics Committee, Oxford University, UK (Refs. 032-07, 06-10 and 164-12) and the Institutional Review Board of the Afghanistan National Public Health Institute, Ministry of Public Health, Afghanistan. In addition trial 1 was approved by the Ethics Committee of the Faculty of Tropical Medicine, Mahidol University, Thailand and trials 2 and 3 were approved by the Ethical Review Committee of the WHO.

## Results

### Trial profile and progress

**Fig. 1:**
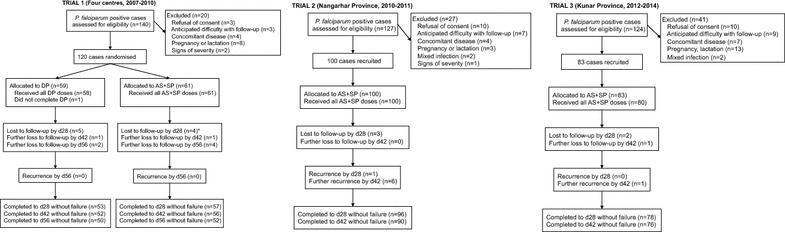
Trial flow. *In trial 1 the four cases lost to follow-up at d28 include one *P. vivax* infection at d28

Trial 1 ran from October 2007 to February 2010. Across the four control centres, 120 cases were enrolled, receiving DP (n = 59) or AS + SP (N = 61); one patient in the DP arm withdrew after two doses of treatment (Fig. 1). Given the clear futility of achieving the planned sample size (550) within a reasonable timeframe, and the evident efficacy of first-line treatment (AS + SP), the trial was stopped and activities shifted to single arm therapeutic efficacy studies aiming to recruit 100 patients each, in line with WHO guidelines [15]. Trial 2 ran from August 2010 to February 2011 in malaria treatment centres in and around Jalalabad. One hundred patients were enrolled and all completed 3 days of treatment. Trial 3 ran from October 2012 to January 2014 in treatment centres in Pech, Shigal, Narang, Khuskunar and Khaskunar districts of Kunar province. 83 patients were recruited over two *P. falciparum* transmission seasons, with three withdrawing before completion of treatment. Given evidence of falling transmission, it was decided not to continue recruitment into a third season. Overall follow-up rates to day 42 were more than 90 % (Fig. 1).

### Baseline characteristics

In trial 1 baseline characteristics were similar between the two treatment groups except for a higher proportion of patients with gametocytes in the AS + SP arm (p = 0.003) (Table 1). Baseline parasitaemia was comparable across all trials.

**Table 1:** Baseline characteristics

Parameter	Study			
	Trial 1		Trial 2	Trial 3
	DP	AS + SP	AS + SP	AS + SP
Year	2007–2010	2007–2010	2010–2011	2012–2014
No. of patients	59	61	100	83
Location (%)				
Jalalabad	41 (69)	42 (69)	100 (100)	–
Takhar	6 (10)	5 (8)	–	–
Faryab	1 (2)	3 (5)	–	–
Kunar	11 (19)	11 (18)	–	83 (100)
Age (years) (median, range)	11 (2–52)	13 (1–70)	15.5 (3–55)	18 (1–74)
Gender male/female (ratio)	43/16 (2.7)	35/26 (1.3)	60/40 (1.5)	49/34 (1.4)
Body weight (kg) (median, range)	31 (8–87)	35 (7.5-85)	50 (10-72)	49 (10–75)
Median parasitaemia/µl (range)	6760 (1220–90,000)	7200 (900–62,160)	4680 (1000–95,000)	3220 (500–59,520)
No. of patients with gametocytes (%)	13 (22)	29 (48)	19 (19)	42 (51)
Mean axillary temp (°C) ± SD	37.9 ± 1.1	37.9 ± 1.0	38.4 ± 0.8	38.4 ± 0.9
Mean hemoglobin (g/dl) ± SD	11.2 ± 1.3	11.2 ± 1.4	11.50 ± 0.9	11.6 ± 1.9
Mean total drug dose (mg/kg) ± SD				
Artemisinin derivative	6.7 ± 1.2 (DHA)	10.42 ± 1.9 (AS)	10.5 ± 2.0 (AS)	10.5 ± 2.4 (AS)
Partner drug	53.9 ± 9.3 (PIP)	27.35 ± 11.9 (SUL)	26.8 ± 5.0 (SUL)	27.3 ± 6.1 (SUL)

### Outcomes

Therapeutic efficacy was high in all the trials, with an ACPR of more than 95 % in all groups and trial stages (Table 2). There were no recrudescences in trials 1 or 3 and only 2 in trial 2, indicating an overall day 42 efficacy of 97.8 % (92.4–99.4). In trial 2 a partial sensitivity analysis (assuming two undefined recurrences to be recrudescences) indicated an ACPR rate of 95.7 % (89.6–98.3 %). No patient deteriorated or developed signs of severity and there were no early treatment failures. There were no serious adverse events and no cases of repeated vomiting of the study medication.

**Table 2:** Efficacy at day 28, day 42 and day 56 (trial 1 only) expressed as percentage with ACPR

	DP	AS + SP T1	AS + SP T2	AS + SP T3
N (enrolled)	59	61	100	83
d28				
Successful follow-up	53	57	97	78
Reinfection	0	0	0	0
Analysed	53	57	97	78
Recrudescence	0	0	1	0
ACPR % (95 % CI)	100 (93.2–100)	100 (93.7–100)	99.0 (94.4–99.8)	100 (95.3–100)
d42				
Successful follow-up	52	56	97	77
Recurrence: reinfection	0	0	3	1
Recurrence: unknown genotype	0	0	2	0
Analysed	52	56	92	76
Recrudescence	0	0	2	0
ACPR (95 % CI)	100 (93.1–100)	100 (93.6–100)	97.8 (92.4–99.4)	100 (95.2–100)
d56				
Successful follow-up	50	52		
Recurrence: reinfection	0	0		
Recurrence: unknown genotype	0	0		
Analysed	50	52		
Recrudescence	0	0		
ACPR (95 % CI)	100 (92.9–100)	100 (93.1–100)		

Successful follow-up indicates patients seen up to and including the corresponding time point. Recurrences due to reinfection and one patient in the AS + SP arm with *P. vivax* infection at d28 were censored from analysis

### Secondary analyses

**Fig. 2:**
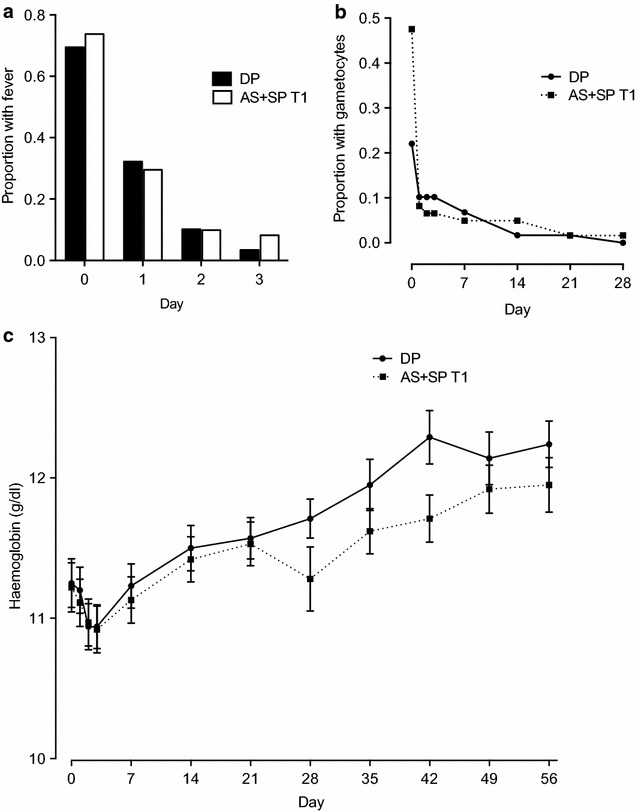
Secondary outcomes in trial 1. Fever (**a**) and gametocyte (**b**) clearance and haemoglobin recovery (**c**) in the two arms of trial 1 (AS + SP vs. DHA-piperaquine)

Parasite clearance was rapid in all trials. Across the three trials, asexual parasites were found at day 1 after treatment in only 18 of 300 patients (6 %) and at day 2 in 3 of 297 patients (1 %) with no patients remaining positive at day 3. In the randomized trial of DP vs. AS + SP, there were no differences between the groups in terms of the proportion of patients with gametocytes or fever (temperature more than 37 °C) after treatment (Fig. 2a, b). Haemoglobin recovery was also similar between the two arms of trial 1 (Fig. 2c).

Mean ± SD day 7 drug levels (adjusted for sample volume) for piperaquine were 98.5 ± 73.3 ng/mL (n = 20 patients), although in two cases levels were less than the limit of detection (0.75 ng/mL). There was a non-significant positive correlation between piperaquine concentration and the body weight-normalized total dose (Spearman r = 0.43, p = 0.06). Day 7 sulphadoxine levels were below the limit of detection (0.5 µg/mL) in nine of the 17 patients studied (spanning a range of ages), a finding that is challenging to correlate with the high measured efficacy.

### Molecular data

**Fig. 3:**
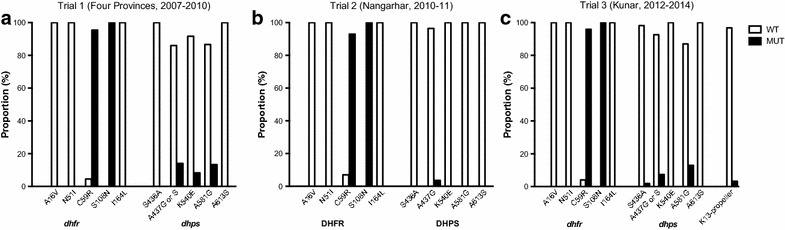
Molecular data. The proportion of wild-type and mutant isolates is shown for five loci in each of *dhfr* and *dhps* (all trials) and the K13 propeller region (trial 3 only). **a**–**c** refer to the three separate trials undertaken, as indicated above each graph

Genotyping for drug-resistance alleles at *dhfr* indicated fixation of the S108 N mutation and that approximately 95 % of parasites carried the C59R mutation (combining all three studies, Fig. 3, Table 3). There were no cases of mutation at *dhfr* A16, N51 or I164 and most parasites were wild-type across the *dhps* gene, with 0.5, 7.7, 2.6 and 10.1 % of isolates carrying mutations at positions 436, 437, 540 and 581 respectively (Table 3). These tended to be combined within haplotypes, with the K540E mutation only found as part of a SGEGA haplotype in trial 1 in one and four isolates from Nangarhar and Kunar provinces respectively. K540E was not seen again in the follow-up trials from either location.

**Table 3:** Molecular findings

Study	Trial 1				Trial 2	Trial 3	Total	
Province	Jalalabad	Takhar	Faryab	Kunar	Jalalabad	Kunar		
DHFR								% with mutation
A16 V	32/0	3/0	2/0	7/0	71/0	49/0	164/0	0
N51I	32/0	3/0	2/0	7/0	71/0	49/0	164/0	0
C59R	2/30	0/3	0/2	0/7	5/66	2/47	9/155	94.5
S108 N	0/32	0/3	0/2	0/7	0/71	0/49	0/164	100.0
I164L	32/0	3/0	2/0	7/0	70/0	49/0	163/0	0
DHPS								
S436A	43/0	6/0	2/0	9/0	83/0	53/1	196/1	0.5
A437G	38/3	6/0	1/1^a^	4/4	80/3	50/4	179/15	7.7
K540E	42/1	6/0	2/0	5/4	83/0	52/0	190/5	2.6
A581G	40/3	6/0	1/1	5/4	34/0	47/7	133/15	10.1
A613S	43/0	6/0	2/0	9/0	34/0	54/0	148/0	0
DHFR haplotype								Proportion (%)
ANC*N*I	2				5	2	9	5.5
AN*RN*I	30	3	2	7	65	47	154	94.5
DHPS haplotype								
SAKAA	38	6	1	4	34	41	124	86.7
SAK*G*A						6	6	4.2
S*GEG*A	1			4			5	3.5
S*G*KAA	1					3	4	2.8
S*G*K*G*A	1					1	2	1.4
S*S*K*G*A			1				1	0.7
*A*AKAA						1	1	0.7

Number of wild-type/mutant samples at five resistance loci in each of the *dhfr* and *dhps* genes, and resulting haplotypes (with mutations in italics). Results indicating mixed wild-type/mutant alleles were counted as mutations. Only haplotypes in which all five loci were successfully determined are described

^a^One sample had the *dhps* A437S mutation

In trial 3, 58 out of 60 samples successfully sequenced at the K13-propeller locus were wild-type; the other samples had a Y493C and a S700L mutation. The patients carrying these isolates had negative blood smears on day 1 (24 h after the start of treatment).

## Discussion

Prospective trials of the clinical efficacy of AS + SP, spanning the decade following its introduction as first-line recommended treatment for *P. falciparum* infection in Afghanistan, provide very reassuring evidence of maintained efficacy with rates of ACPR well over 95 % in all studies. Accompanying assessments of molecular markers explain these clinical responses. As in the rest of Asia and much of sub-Saharan Africa, two polymorphisms in *dhfr* (59R and 108 N) remain at or near fixation, but no other *dhfr* mutations are found in Afghanistan. A previous report generated concerns over the prevalence of the *dhps* triple mutation 437G/540E/581G in Nangarhar and Kunar provinces (the molecular data for trial 1 of this paper) [23] but the subsequent data indicate no evidence of this triple mutation in either province, and there was no increase in the individual mutations that make up the haplotype. In summary, there is no phenotypic or genotypic evidence of worsening resistance to SP a decade after the introduction of AS + SP in Afghanistan.

Compared to this, neighbouring countries show relatively more concerning resistance marker profiles. Surveys from Pakistan within the last decade show significant numbers of parasites mutated at DHPS 437G [26–28] as well as 540E and 581G (generally in mixed infections) [28]. Studies in central India [29, 30] and Iran [31, 32] also describe increasing mutations at 437G. However, AS + SP treatment of *P. falciparum* does not yet appear to have been significantly compromised by molecular changes in any of these locations. Only in northeast India is there evidence for a high prevalence of ‘quintuple’ mutation haplotypes based on these two genes [33] in association with reports of failure of AS + SP.

This is the first study to examine the K13 artemisinin resistance marker in Central Asia. Only two samples (less than 5 %) contained a mutation in the propeller domain; Y493C and S700L. Neither mutation has been previously reported, although a different mutation at 493, Y493H, is common and causally linked to artemisinin resistance in Cambodia [24, 34]. The patients carrying these isolates had rapid parasite clearance. Overall, the data indicate that isolates remain fully sensitive to artemisinins in Afghanistan.

The maintained efficacy of AS + SP, with no evidence of adaptive evolution in the *dhps* gene or increasing mutations in *dhfr*, is to some degree reminiscent of the return of in vitro mefloquine sensitivity following addition of artesunate to mefloquine at the Thai–Myanmar border in the 1990s [35]. While use of anti-malarials as ACT is predicted to protect against worsening resistance [36], it is not clear why AS + SP has retained its efficacy in Afghanistan over the course of 10 years despite a degree of pyrimethamine resistance at the outset, the lack of an available coformulation and clear evidence of worsening sensitivity in neighbouring countries. Factors that may have contributed to the maintained efficacy include restriction in terms of access to anti-malarials, a lack of fake or substandard anti-malarials and human factors, such as a tendency to complete prescribed courses.

## Conclusions

AS + SP, the first-line treatment for falciparum malaria in Afghanistan, remains efficacious therapy for uncomplicated falciparum malaria, both in terms of initial parasite clearance and cure. Accompanying molecular data indicate a stable situation with regard to SP resistance, and suggest that AS + SP is likely to retain a high level of efficacy as treatment for *P. falciparum* for a substantial period of time as Afghanistan moves towards elimination.
